# Molecular and Toxicity Analyses of White Granulated Sugar and Other Processing Products Derived From Transgenic Sugarcane

**DOI:** 10.3389/fpls.2020.596918

**Published:** 2020-11-26

**Authors:** Wenzhi Wang, Benpeng Yang, Juangang Wang, Xiaoyan Feng, Cuilian Feng, Tingting Zhao, Linbo Shen, Qinnan Wang, Zhuandi Wu, Shuzhen Zhang, Zhengqiang Ma

**Affiliations:** ^1^Crop Genomics and Bioinformatics Center and National Key Laboratory of Crop Genetics and Germplasm Enhancement, Nanjing Agricultural University, Nanjing, China; ^2^Institute of Tropical Bioscience and Biotechnology, Chinese Academy of Tropical Agricultural Sciences, Haikou, China; ^3^Institute of Bioengineering, Guangdong Academy of Sciences, Guangzhou, China; ^4^Sugarcane Research Institute, Yunnan Academy of Agricultural Sciences, Kaiyuan, China

**Keywords:** toxicity feeding bioassay, enzyme-linked immunosorbent assay, real-time fluorescent quantitative PCR, PCR analysis, genetic modification sugar, genetic modification sugarcane

## Abstract

This study aimed to prepare the sugar industry for the possible introduction of genetically modified (GM) sugarcane and derived retail sugar products and to address several potential public concerns regarding the characteristics and safety of these products. GM sugarcane lines with integrated *Cry1Ab* and *EPSPS* foreign genes were used for GM sugar production. Traditional PCR, real-time fluorescent quantitative PCR (RT-qPCR), and enzyme-linked immunosorbent assay (ELISA) were performed in analyzing leaves, stems, and other derived materials during sugar production, such as fibers, clarified juices, filter mud, syrups, molasses, and final GM sugar product. The toxicity of GM sugar was examined with a feeding bioassay using *Helicoverpa armigera* larvae. PCR and RT-qPCR results showed that the leaves, stems, fibers, juices, syrups, filter mud, molasses, and white granulated sugar from GM sugarcane can be distinguished from those derived from non-GM sugarcane. The RT-qPCR detection method using short amplified product primers was more accurate than the traditional PCR method. Molecular analysis results indicated that trace amounts of DNA residues remain in GM sugar, and thus it can be accurately characterized using molecular analysis methods. ELISA results showed that only the leaves, stems, fibers, and juices sampled from the GM sugarcane differed from those derived from the non-GM sugarcane, indicating that filter mud, syrup, molasses, and white sugar did not contain detectable Cry1Ab and EPSPS proteins. Toxicity analysis showed that the GM sugar was not toxic to the *H. armigera* larvae. The final results showed that the GM sugar had no active proteins despite containing trace amounts of DNA residues. This finding will help to pave the way for the commercialization of GM sugarcane and production of GM sugar.

## Introduction

Transgenic crops, such as soybean (Pallett, 2018; Tian et al., 2019), corn (Li et al., 2018), rapeseed (Zhang et al., 2020), and cotton (Chen et al., 2019) are cultivated to improve crop yield and resistance to biotic and abiotic stresses. Transgenic crops have greatly contributed to meet the increasing demand for food by the growing world population (Aldemita et al., 2015; Koch et al., 2015; Giraldo et al., 2019). In 2018, a total of 70 countries have planted or imported genetically modified (GM) crops or products, and 26 countries have planted nearly 0.2 billion ha of GM crops (International organization for the application of agricultural biotechnology services (ISAAA), 2019). The annual planting of GM crops has resulted in huge economic gains worldwide (Brooks and Barfoot, 2014). With the development of plant biotechnology, the diversity of GM crops has increased. However, few transgenic crop varieties have been commercialized since the first reported transgenic plant in the 1990s (Bruening and Lyons, 2000) owing to safety concerns and unfavorable reception from consumers (Lucht, 2015). The first transgenic sugarcane was generated in 1992 by Bower and Brich in Australia (Bower and Brich, 1992). Since then, a lot of transgenic sugarcane cases with improved disease (Guo et al., 2015; Yao et al., 2017), pest (Gao et al., 2018; Zhou et al., 2018), herbicide resistance (Wang et al., 2017a, b), and other traits have been generated (Hoang et al., 2015; Ramiro et al., 2016). In 2017, the commercial use of transgenic sugarcane with the *Bt* foreign gene has been permitted in Brazil (International organization for the application of agricultural biotechnology services (ISAAA), 2018). Sugarcane is an important original raw material for white granulated sugar production. With the initial permission for the actual planting of the first GM sugarcane, safety concerns regarding GM sugar have been raised by consumers worldwide. In this study, we produced GM sugar from GM sugarcane lines under laboratory conditions by using methods that emulate factory production processes. Original material GM sugarcane lines integrated with the *Cry1Ab* and *EPSPS* foreign genes were obtained in our previous research (Wang et al., 2017a). *Cry1Ab* and *EPSPS* genes had been widely used in crop genetic improvement and produced herbicide and insect resistance crops (Shu et al., 2017; Chinnadurai et al., 2018; Fartyal et al., 2018; Fu et al., 2018). And more over crops integrated with *Cry1Ab* and *EPSPS* genes are the main GM crops, which had been permitted for commercialized use. The quality of the GM sugar was analyzed and compared with that of non-GM sugar bought from a supermarket. During GM sugar production, derived materials, fibers (after the crushing and extraction of juices), clarified juices (standing and upper supernatants of juices), syrup (after the boiling and concentration of clarified juices), filter mud (impurity during heating and concentration of clarified juices), molasses (segregated through the crystallization of white granulated sugar), and final white granulated sugar (crystallized from consistent concentration of syrups) were sampled for DNA extraction and molecular analysis. Detectable Cry1Ab and EPSPS proteins of all the samples were examined by ELISA. The toxicity of the GM sugar was examined by performing a feeding bioassay on *Helicoverpa armigera* larvae. All results showed that GM sugarcane can be safely commercialized and used in producing GM sugar.

## Materials and Methods

### Plant Material

GM sugarcane was produced using the *Agrobacterium*-mediated transformation method in our previous research (Wang et al., 2017a). The foreign insecticidal Bt gene *Cry1Ab*, glyphosate tolerant gene *EPSPS*, and selection marker gene *PMI* (Hu et al., 2016) were integrated into the GM sugarcane genome. The GM sugarcane was planted in a field for approximately 11 months and harvested after maturation. The harvested GM sugarcane was used in producing GM sugar.

### Production of GM Sugar

GM sugar was produced in the laboratory through a method that emulates factory production. This work was accomplished by the ***Sugarcane Research Institute of Yunnan Academy of Agricultural Sciences in Kaiyuan, Yunnan, China***. The production protocol for white granulated sugar is not shown because the Institute owns the patent. Derived materials, fibers (after the crushing and extraction of juices), clarified juices (standing and upper supernatants of the juices), syrups (after boiling and concentration of the clarified juices), filter mud (impurity during the heating and concentration of clarified juices), molasses (segregated from the crystallization of the white granulated sugar), and final white granulated sugar product (GM sugar) were sampled during the processing.

### Quality Analysis of White Granulated Sugar

We analyzed the quality of the GM sugar product to ensure that the laboratory-produced GM sugar was sufficiently purified and can be used in this research. Quality analysis was accomplished by the ***Detection and Standard Research Center of Guangdong Bioengineering Institute in Guangzhou, Guangdong, China***. The quality index of the GM sugar was compared with those of the non-GM sugar and Chinese national standard for sugar products (GB/T 317-2018).

### DNA Extraction of All Derived Products

The leaves, stems, fibers, clarified juices, filter mud, syrups, molasses, and GM sugar derived from the GM sugarcane and non-GM sugar bought from a supermarket were sampled for DNA extraction. Each sample had three replicates.

DNA was extracted from the leaves, stems, fibers, juices, and filter mud as follows: the solid samples of leaves (50 mg), stems (200 mg), and fibers (25 mg) were cut into small pieces and ground into powder with liquid nitrogen. The liquid samples (200 mg or 200 μl) of juices and filter mud were sampled for DNA extraction. All the samples were transferred to 2 ml centrifuge tubes. Then, 800 μl of CTAB lysis buffer was added to each tube and heated to 65°C for 30 min. The tubes were then rolled gently two or three times during incubation. After incubation, the lysis buffer was cooled down to room temperature, and then 1 ml of a solution containing chloroform and isoamyl alcohol in a ratio of 24:1 was added to each tube and mixed gently. The mixture was centrifuged, and 700 μl of the supernatant was transferred to a new 1.5 ml centrifuge tube. DNA was precipitated with 700 μl of isopropanol, washed twice with 70% alcohol, dried under room temperature, and finally dissolved with 50 μl of sterile water. Then, 2 μl of the extracted DNA was detected using a gel imaging analysis system. The concentrations of all DNA samples were measured using UV absorbance. The DNA was used as the template for traditional PCR and real-time fluorescence quantitative PCR (RT-qPCR) assays.

DNA was extracted from the syrups and molasses as follows: syrup and molasses samples (5 ml) were transferred to 50 ml centrifuge tubes, and each was mixed with 20 ml of CTAB lysis buffer. The mixtures were heated to 65°C and maintained for 30 min in an incubator. The tubes were rolled gently two or three times during incubation. After incubation, all the extraction buffers were cooled down to room temperature, and then 25 ml of the chloroform-isoamyl alcohol (24:1) mixture was added to each tube and mixed gently. The mixture buffer was centrifuged, and 20 ml of the supernatant was transferred to a new 50 ml centrifuge tube. DNA was precipitated with 20 ml of isopropanol. The mixture was centrifuged, and the DNA precipitate was dissolved in 1 ml of sterile water. Then, 1 ml of the DNA solution was collected, poured into a 2.0 ml centrifuge tube, and mixed with 100 μl of 3 M NaAc. DNA was precipitated again with 1 ml of isopropanol, washed twice with 70% alcohol, dried at room temperature, and finally dissolved with 50 μl of sterile water. Then, 2 μl of the extracted DNA was detected using the gel imaging analysis system. The concentrations of all DNA samples were measured using UV absorbance. The DNA was used as the template for traditional PCR and RT-qPCR.

### DNA Extraction of White Granulated Sugar

The laboratory-produced GM sugar and commercial non-GM sugar were sampled for DNA extraction. Each sample had three replicates. DNA was extracted as follows: the two kinds of sugar (5 g) were transferred to 50 ml centrifuge tubes and mixed with 20 ml of CTAB lysis buffer. The tubes were heated to 65°C and maintained for 60 min in the incubator. The tubes were shook, and the sugar in the lysis buffer was completely dissolved before the end of the incubation period. The lysis buffer was cooled down to room temperature, and 25 ml of chloroform:isoamyl-alcohol (24:1) mixture was added to each tube and mixed gently. The mixture buffer was centrifuged, and 25 ml of the supernatant was transferred to a new 50 ml centrifuge tube. Chloroform:isoamyl alcohol (24:1; 25 ml) was again added to each tube and mixed gently. The tubes were recentrifuged, and 20 ml of the supernatant was collected and poured into a new 50 ml centrifuge tube. The DNA was precipitated with 20 ml of isopropanol. The tube was centrifuged, and the DNA precipitate was dissolved with 1 ml of sterile water. Approximately 1 ml of the DNA solution was transferred to a 2.0 ml centrifuge tube and mixed with 100 μl of 3 M NaAc. The DNA was reprecipitated with 1 ml of isopropanol, washed twice with 70% alcohol, dried at room temperature, and finally dissolved with 50 μl of sterile water. Then, 2 μl of the extracted DNA was detected using the gel imaging analysis system. The concentrations of all the DNA samples were measured using UV absorbance. The DNA was used as the template for traditional PCR and RT-qPCR.

### PCR Primer Designed for the Endogenous and Exogenous Genes

According to the sequences of the endogenous gene *actin* and exogenous genes *PMI*, *EPSPS*, and *Cry1Ab*, four pairs of primers for each gene were designed using Primer Premier 5 for the traditional PCR assay or RT-qPCR assay. The primer sequences are shown in Table 1.

**Table 1:** Primer sequences for the amplification of endogenous and exogenous genes.

Gene	Size of PCR Products (bp)	Primer sequence	
Actin	112	Forward	CTGGAATGGTCAAGGCTGGT
		Reverse	TCCTTCTGTCCCATCCCTACC
PMI	142	Forward	CTGACCCCCAAGTACATCGAC
		Reverse	TGAAGGCGAAGTCGTCCAC
EPSPS	130	Forward	AAGACGCCTAACCCGATCAC
		Reverse	TATGATCGCGGGTCAACACC
Cry1Ab	104	Forward	TGATCGGCAACTACACCGAC
		Reverse	GCGGAACTGGTTGTACCTGA

All primers for PCR analysis were designed with Primer Premier version 5.0. Amplified 112 bp DNA fragment for the endogenous *actin* gene, 142 bp DNA fragment for the *PMI* gene, 130 bp DNA fragment for the *EPSPS* gene, and 104 bp DNA fragment for the *Cry1Ab* gene. Each pair of primers shares the same TM value (58°C) for PCR analysis.

### Traditional PCR Analysis of All Samples

Extracted DNA from each kind of sample was used for traditional PCR. Each 25 μl of the traditional PCR reactant contained 12.5 μl of PCR Master Mix buffer (2×), 2 μl of each kind of template DNA, 1 μl of forward primer, 1 μl of reverse primer, and 8.5 μl of double-distilled (dd) H_2_O. PCR was conducted as follows: initial polymerase activation at 105°C for 5 min; 35 cycles at 95°C for 30 s, 58°C for 30 s, 72°C for 30 s, and final extension at 72°C for 10 min. PCR products were detected on 1.0% (w/v) agarose gel, and false positive results were prevented by repeating PCR analysis at least three times. A positive sample was defined as the sample that showed two positive amplifications. Final results were then recorded in a table.

### RT-qPCR of All Samples

DNA extractions from the leaves, stems, and fibers were diluted 10 times with dd H_2_O and then sampled for RT-qPCR. DNA extractions from the juices, syrup, filters mud, molasses, and two white granulated sugars (GM and non-GM sugar) were sampled directly without dilution for RT-qPCR. Each 10 μl of the RT-qPCR reactant contained 5 μl of RT-qPCR mix buffer (2×), 2 μl of each kind of template DNA, 0.2 μl (10 μM) of each forward and reverse primer, and 2.6 μl of dd H_2_O. The PCR reaction was conducted as follows: initial polymerase activation at 95°C for 5 min, then 40 cycles at 95°C for 30 s, 58°C for 30 s, and 72°C for 30 s. The results were evaluated based on the CT value and dissociation curve. A CT value that is less than 38 and has the same dissociation curve as that of the positive control (CK+) was defined as positive amplification. A positive sample was defined as the sample that showed two positive amplifications. The final results were then recorded in a table.

### ELISA of All Samples

ELISA kits (Bt-Cry1Ab/1Ac ELISA Kit, Agdia, United States; CP4-EPSPS ELISA kit, Agdia, United States) were used in characterizing Cry1Ab and EPSPS protein residues in all samples, and the ELISA was performed three times for each sample. Purified EPSPS and Cry1Ab proteins from ELISA kits were set as the positive controls (CK+). ELISA reactions were performed mainly according to the instructions in the ELISA kits. Different kinds of samples were prepared for ELISA as follows: 0.1 g of leaves, stems, or fibers was ground into powder with liquid nitrogen and added to the bottom of 1.5 ml Eppendorf tubes. The pestles from the kit were inserted into each tube, and solid samples were ground by rotating the pestles against the sides of the tube with twisting motions. This process was continued until the stem tissue samples were ground well. The liquid samples: juices, syrups, filter mud, and molasses (100 μl) were collected and added directly to 1.5 ml Eppendorf tubes. All well-ground solid and liquid samples were then mixed with 1 ml of 1 × phosphate-buffered saline with Tween 20 (PBST) buffer. GM and non-GM white granulated sugars (100 mg) were added to 1.5 ml Eppendorf tube and diluted directly with 1 ml of PBST buffer. The mixtures were mixed well by rotating the pestles in the tubes or gently placing the tubes upside down. All tubes with the sample mixtures were centrifuged for 1 min, and the liquid supernatants were used for ELISA. ELISA was conducted using an ELISA kit according to the manufacturer’s instructions. The development of blue color in the ELISA mixture indicated the presence of target proteins. False positive results were prevented by performing ELISA analysis at least three times. In three replications, a sample with two ELISA positive results was defined as a positive sample. The final results were then recorded in a table.

### Toxicity Analysis of GM White Sugar

*Helicoverpa armigera* larvae were used in testing the toxicity of the GM sugar and determining whether the sugar still contained active Cry1Ab protein. *Helicoverpa armigera* larvae with the same size (provide by ***Hubei Academy Agriculture Sciences, China***) were selected for the feeding bioassay. All larvae were starved for 12 h before the bioassay. Briefly, 4 g of GM or non-GM sugar was sampled and mixed with 40 ml of larva medium (8 g of larva fodder powder +40 ml of ddH_2_O + 0.35 g of agar). The larva fodder mixture was equally distributed to a 12-well cell culture plate (Eppendorf, Germany). Transgenic sugarcane (original material for GM sugar production) stem material (4 g) containing active Cry1Ab protein was sampled and ground into powder with liquid nitrogen. The powder was then mixed with 40 ml of larva medium. This mixture medium was set as the toxicity positive control. Twelve larvae with the same body size and weight were distributed to the wells. The culture plates were covered and placed in an incubator in the dark at 28 ± 2°C and 70% relative humidity. All feeding bioassays were conducted for 3 weeks. The body weight of each larva was recorded from the 3rd day to the 10th day. The average weights and standard deviations of the 12 larvae fed with different media were analyzed using Excel version 2007. The development and pupation of larvae were observed during these 3 weeks.

## Results and Discussion

### Quality Analysis of GM and Non-GM Sugar

The laboratory-produced GM sugar and the commercial non-GM sugar were sampled and sent to the ***Detection and Standard Research Center of Guangdong Bioengineering Institute, China*** for quality analysis. The quality of the GM sugar was compared with that of the non-GM sugar and the Chinese national standard for sugar product quality (GB/T 317-2018). Table 2 shows that the laboratory-produced GM sugar had better quality than the commercial non-GM sugar. Moreover, the ***reducing sugar content*, c*onductance ash content***, and ***loss on drying content*** of the GM sugar were better than the purity grade of the Chinese national standard, and its **s*ucrose content*, *turbidity***, and ***insoluble impurity content*** were better than the superior grade. The color value was better than the first grade. Except ***loss on drying content***, other the indices of the GM sugar were better than those of the non-GM sugar. Thus, the laboratory-produced GM sugar was pure enough and better than the commercial non-GM sugar. The laboratory-produced GM sugar can be utilized for molecular analysis in this research.

**Table 2:** Quality analysis of GM and non-GM sugar.

Test item	National standard (China) GB/T 317-2018			Sugar (GM)	Sugar (Non-GM)
	Pure	Senior	First grade		
Sucrose content (g/100 g) ≥	99.8	99.7	99.6	99.78^**^	99.6^*^
Reducing sugar content (g/100 g) ≤	0.03	0.04	0.1	0.010^***^	0.056^*^
Conductance ash content (g/100 g) ≤	0.02	0.04	0.1	0.017^***^	0.036^**^
Loss on drying content (g/100 g) ≤	0.05	0.06	0.07	0.023^***^	0.020^***^
Color value/IU ≤	25	60	150	80^*^	**201**
Turbidity/MAU ≤	30	80	160	31^**^	69^**^
Insoluble impurities content/(mg/kg) ≤	10	20	40	15^**^	16^***^
SO_2_/(mg/kg) ≤	100			**3.2**	**3.2**

IU, international unit; MAU, milli absorbance unit.

* Better than the first grade;

** Better than the senior grade;

*** Better than the purify grade.

### Concentrations of All DNA Samples

**Figure 1:**
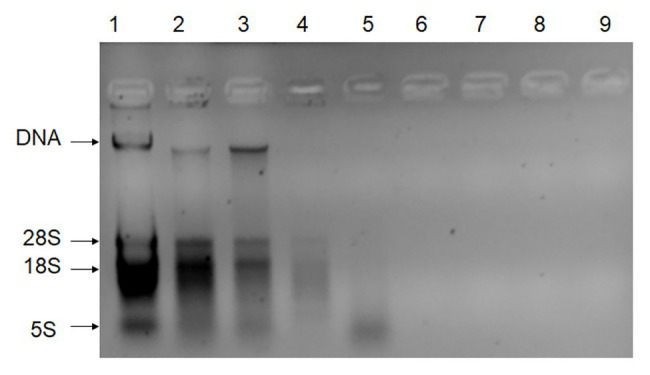
Gel imaging analysis of all DNA extractions (without the digestion of RNA). Lane 1: DNA extracted from leaves; Lane 2: DNA extracted from stems; Lane 3: DNA extracted from fibers; Lane 4: DNA extracted from juices; Lane 5: DNA extracted from filter mud; Lane 6: DNA extracted from the syrup; Lane 7: DNA extracted from molasses; Lane 8: DNA extracted from the GM sugar; Lane 9: DNA extracted from the non-GM sugar.

DNA extracts (without the digestion of RNA) from the leaves, stems, fibers, juices, filter mud, syrup, molasses, GM sugar, and non-GM sugar were measured with the gel imaging analysis system (Figure 1) on the basis of UV absorbance. DNA extraction from the leaves, stems, and fibers was observed with the gel imaging analysis system. DNA extracted from the juices, filter mud, syrup, molasses, GM sugar, and non-GM sugar had extremely low concentrations or degraded poorly. Thus, DNA from these samples was hardly observed in the gel imaging analysis system. The exact DNA concentration was measured on the basis of UV absorbance, and the results are shown in Table 3. DNA from the leaves, stems, and fibers was obtained normally and easily using simple DNA extraction protocols. Juices were derived from the crushed sugarcane stalk and contained small amounts of cells. Thus, the total DNA from juices was obtained easily with the CTAB method. We obtained 7.8 μg of DNA from each milliliter of juice. Filter mud is an impurity generated during the heating and concentration of clarified juice. Thus, this by-product contains numerous cells, and its DNA can be extracted easily. We obtained 52.9 μg of DNA from each milliliter of filter mud. After filtration, boiling, concentration, and crystallization, clarified juice turned into syrup. Most cells were filtered out, leaving small amounts of cells in the syrup. However, these cells were destroyed, and DNA degraded poorly because of the boiling and concentration treatments. Thus, DNA from the syrup was very low and difficult to extract. A considerable amount of syrup was needed to enrich combined DNA. In this research, we sampled 5 ml each of syrup for DNA extraction. We obtained 0.084 μg of DNA from each milliliter of syrup. Molasses are segregated from the crystallization of white granulated sugar and contain some kinds of cell impurities. Thus, these compounds contain more DNA than syrup and white granulated sugar. We obtained 0.21 μg of DNA from each milliliter of molasses. White granulated sugar is produced through the crystallization of highly concentrated syrup and is a kind of purified product. Thus, DNA in white granulated sugar is reduced and degrades poorly. A substantial amount of sample is needed to enrich DNA during extraction. The amount of DNA remaining in white granulated sugar mainly depends on the purity grade of white granulated sugar. In this research, we sampled 5 g of white granulated sugar for DNA extraction. We obtained only approximately 0.021 and 0.032 μg of DNA from each gram of GM and non-GM sugar, respectively.

**Table 3:** DNA concentrations of all samples.

Item	Leaf (GM)	Stem (GM)	Fiber (GM)	Juice (GM)	Filter mud (GM)	Syrup (GM)	Molasses (GM)	Sugar (GM)	Sugar (Non-GM)
Weight of sample for DNA extraction	50 mg	200 mg	25 mg	200 μl	200 μl	5 ml	5 ml	5 g	5 g
Final volume of DNA dilution (ml)	0.05	0.05	0.05	0.05	0.05	0.05	0.05	0.05	0.05
Concentration of final DNA dilution (μg/ml)	1109.3 ± 29.4	379.3 ± 17.0	287.0 ± 17.4	31.1 ± 6.1	211.7 ± 7.6	8.4 ± 0.5	20.9 ± 1.9	2.0 ± 0.5	3.1 ± 0.8
Weight of total DNA extracted (μg)	56.08 ± 1.45	18.97 ± 0.85	14.35 ± 0.87	1.56 ± 0.31	10.58 ± 0.38	0.42 ± 0.03	1.05 ± 0.09	0.11 ± 0.03	0.16 ± 0.04
DNA content from per unit of sample (μg/g)	1109.3 ± 29.4	94.8 ± 4.3	574.0 ± 34.9	7.8 ± 1.5	52.9 ± 1.9	0.084 ± 0.005	0.21 ± 0.02	0.021 ± 0.005	0.032 ± 0.007

Weight of total DNA extracted = Concentration of final DNA dilution × Final volume of DNA dilution;

DNA content from per unit of sample = Weight of total DNA extracted/Weight of sample for DNA extraction.

### Traditional PCR Analysis of All Samples

**Figure 2:**
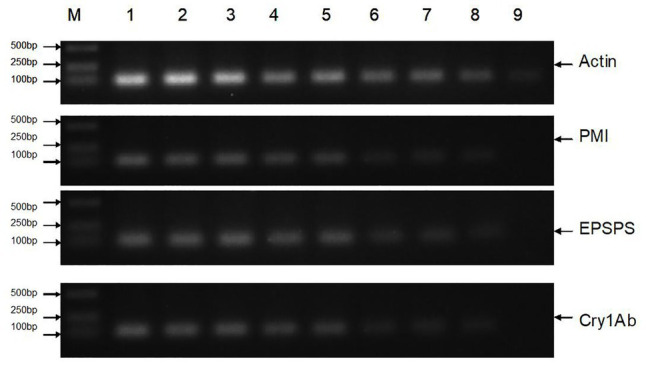
Traditional PCR analysis of all samples. Lane M: DNA Marker; Lane 1: Leaf (GM); Lane 2: Stem (GM); Lane 3: Fiber (GM); Lane 4: Juice (GM); Lane 5: Filter mud (GM); Lane 6: Syrup (GM); Lane 7: Molasses (GM); Lane 8: Sugar (GM); Lane 9: Sugar (Non-GM).

All DNA extracts were sampled for traditional PCR analysis (Figure 2, only part of the images are shown), and the results are presented in Table 4. The extraction process was repeated three times for each sample. The results of the traditional PCR showed that the DNA samples from the transgenic sugarcane leaves, stems, fibers, juices, filter mud, syrups, and molasses were determined easily. The images in the agarose gels for syrups and molasses showed weakly amplified fragments but were confirmed to be positive amplifications. DNA samples from the GM and non-GM sugar had extremely low concentrations and degraded poorly. Thus, DNA was difficult to characterize through traditional PCR. Some repeats of PCR action produced dark and weak bands on the image. Thus, whether the bands were positive amplifications was difficult to determine. For the traditional PCR, a more efficient DNA collection method is required, and more DNA fragments should be enriched from white granulated sugar.

**Table 4:** PCR analysis of all DNA samples.

Item	Leaf (GM)	Stem (GM)	Fiber (GM)	Juice (GM)	Filter mud (GM)	Syrup (GM)	Molasses (GM)	Sugar (GM)	Sugar (Non-GM)
Actin	+++	+++	+++	+++	+++	+++	+++	−++	++−
PMI	+++	+++	+++	+++	+++	+++	+++	+ − -	---
EPSPS	+++	+++	+++	+++	+++	+++	+++	+ − -	---
Cry1Ab	+++	+++	+++	+++	+++	+++	+++	+ − -	---

Each sample repeat three times; +: PCR positive; −: PCR negative.

### RT-qPCR Analysis of All Samples

**Figure 3:**
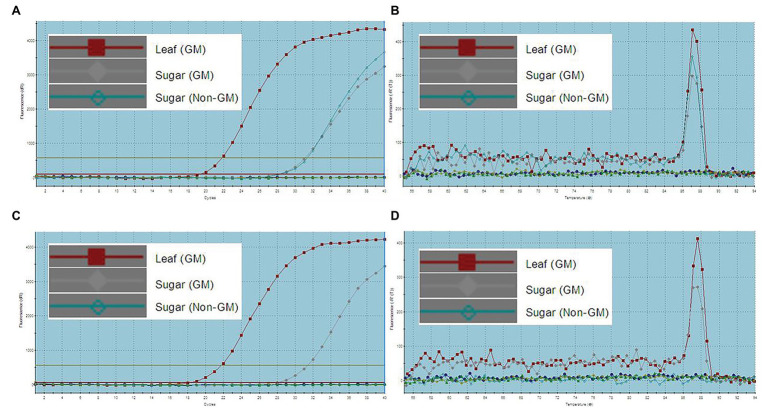
RT-qPCR analysis of GM and non-GM sugar. **(A)**: amplification plots of endogenous gene *actin* (CT value: GM leaf, 22.12; GM sugar, 31.42; non-GM sugar, 31.17); **(B)**: dissociation curve of endogenous gene *actin*, the three samples share the same dissociation curve; **(C)**: amplification plots of the endogenous gene *Cry1Ab* (CT value: GM leaf, 21.81; GM sugar, 31.44; non-GM sugar, no CT value); **(D)**: dissociation curve of the endogenous gene Cry1Ab, GM leaf sample and GM sugar share the same dissociation curve, but the non-GM sugar has no dissociation curve.

All DNA extracts were sampled for RT-qPCR with the same primers used in traditional PCR analysis. The results were evaluated based on the CT value and dissociation curve. A CT value of less than 35 and with dissociation curve similar to that of CK+ (GM leaf sample) was defined to be positive amplification. The final results in Table 5 show that not only the leaves, stems, fibers, juices, filter mud, syrups, and molasses from transgenic sugarcane were defined, but also GM sugar was defined as well. Figure 3A shows the amplification plots of the endogenous gene *actin* RT-qPCR analysis of CK+ (GM leaf sample), GM sugar, and non-GM sugar. The CT values of these samples were less than 35. Figure 3B shows that the three samples shared the same dissociation curve. Thus, the samples were positively amplified of endogenous gene actin by RT-qPCR analysis. Figure 3C shows the amplification plots of the foreign gene *Cry1Ab*, which were obtained by conducting RT-qPCR analysis on CK+ (GM leaf sample), GM sugar, and non-GM sugar. The CT values of CK+ (GM leaf sample) and GM sugar were less than 35, but non-GM sugar showed no CT value. Figure 3D shows that CK+ (GM leaf sample) and GM sugar shared the same dissociation curve, but the non-GM sugar had no dissociation curve. Thus, it is a positive amplification of CK+ (GM leaf sample) and GM sugar of the foreign gene *Cry1Ab*, but it is the failed amplification of non-GM sugar. It presented the same amplification result obtained from the other two foreign genes *PMI* and *EPSPS* (Figures are not shown). Thus, for the GM sugar, all the three foreign genes and endogenous gene *actin* were positively amplified. For the non-GM sugar, only *actin* was positively amplified. All the three foreign genes were negative in the non-GM sugar. Thus, the results showed that GM sugar can be distinguished from the non-GM sugar through RT-qPCR. For accurate GM sugar RT-qPCR analysis results, efficient DNA collection methods should be used in enriching DNA residues from GM sugar.

**Table 5:** RT-qPCR analysis of all DNA samples.

Item	Leaf (GM)	Stem (GM)	Fiber (GM)	Juice (GM)	Filter mud (GM)	Syrup (GM)	Molasses (GM)	Sugar (GM)	Sugar (Non-GM)
Actin	+++	+++	+++	+++	+++	+++	+++	+++	+++
PMI	+++	+++	+++	+++	+++	+++	+++	+++	---
EPSPS	+++	+++	+++	+++	+++	+++	+++	+++	---
Cry1Ab	+++	+++	+++	+++	+++	+++	+++	+++	---

Each sample repeat three times; +: RT-qPCR positive; −: RT-qPCR negative.

### ELISA of All Samples

**Figure 4:**
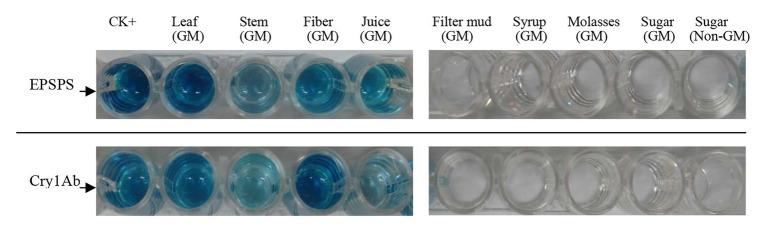
ELISA of all samples.

Whether detectable EPSPS and Cry1Ab proteins were present in all samples were determined using ELISA, which was performed three times for each sample. Purified EPSPS and Cry1Ab proteins from ELISA kits (Bt-Cry1Ab/1Ac ELISA kit, Agdia, United States; CP4-EPSPS ELISA Kit, Agdia, United States) were set as positive controls (CK+). The development of blue color as the positive control in the ELISA reaction buffer indicates the presence of the target proteins of the *Cry1Ab* or *EPSPS* gene (Figure 4). Table 6 shows that the leaves, stems, fibers, and juices from the transgenic sugarcane were positive for Cry1Ab and EPSPS proteins. Meanwhile, the filter mud, syrups, molasses, and white granulated sugar (GM and non-GM sugar) were negative. Filter mud, syrups, molasses, and white granulated sugar were derived after boiling, concentration, and filtration treatment of clarified juice. Thus, all the proteins in these samples were denatured because of the heating treatment, and the samples from the filter mud, syrups, molasses, and GM sugar showed negative ELISA results.

**Table 6:** ELISA results of all samples.

Item	Leaf (GM)	Stem (GM)	Fiber (GM)	Juice (GM)	Filter mud (GM)	Syrup (GM)	Molasses (GM)	Sugar (GM)	Sugar (Non-GM)
EPSPS	+++	+++	+++	+++	---	---	---	---	---
Cry1Ab	+++	+++	+++	+++	---	---	---	---	---

Each sample repeat three times; +: ELISA positive; −: ELISA negative.

### Toxicity Analysis of GM Sugar

**Figure 5:**
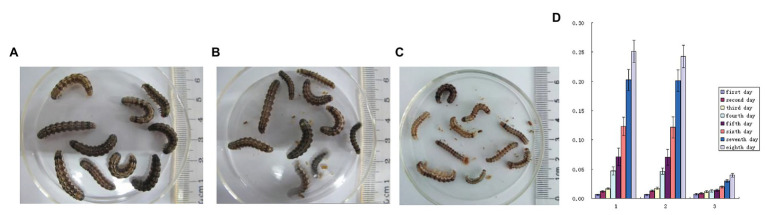
Larvae feeding bioassay. **(A)**: larvae feed with larva fodder mix with GM sugar; **(B)**: larvae feed with larva fodder mix with non-GM sugar; **(C)**: larvae feed with larva fodder mix with GM sugarcane stem material. **(D)**: 1: Daily average weight of 10 larvae fed with larva fodder mix with GM sugar; 2: Daily average weight of 10 larvae fed with larva fodder mix with non-GM sugar; 3: Daily average weight of 10 larvae fed with larva fodder mix with GM sugarcane stem material.

GM sugar was produced from the *Cry1Ab* gene transgenic sugarcane. For the evaluation of GM sugar toxicity, 4 g of GM sugar was mixed with 40 ml of larva fodder medium and used for the feeding bioassay. Non-GM white sugar (4 g) was set as the negative control, and 4 g of transgenic sugarcane stem material was set as the positive control. The results showed that, compared with the larvae fed with non-GM sugar (Figure 5B), the larvae fed with GM sugar (Figure 5A) grew and developed normally. No remarkable differences in body weight, pupation, and eclosion were found in the larvae fed by the two kinds of white granulated sugar. The average weight of the larvae fed with GM sugar increased from 0.006192 ± 0.001158 g to 0.250758 ± 0.019165 g in the first 8 days (Figure 5D). The average weight of the larvae fed with non-GM sugar increased from 0.00605 ± 0.001236 g to 0.242425 ± 0.019447 g in the first 8 days. In addition, all the larvae pupated and underwent eclosion. However, the larvae fed with transgenic sugarcane stem were weak and small (Figure 5C). The average weight of larvae fed with transgenic sugarcane stem increased from 0.0071 ± 0.001183 g to 0.039508 ± 0.003321 g in the first 8 days. Moreover, pupation and eclosion were delayed remarkably compared with those in the larvae fed with other two kinds of white granulated sugar. This result was in accordance with our previous toxicity assay on transgenic sugarcane (Wang et al., 2017a). Thus, the final result of the toxicity assay shows that the nontoxicity of GM sugar to the larvae is primarily due to the absence of active Cry1Ab protein in the GM sugar.

## Discussion

In this study, we mimicked the factory production process to produce transgenic GM sugar. The quality of the GM sugar was analyzed and compared with that of commercial sugar and the Chinese national standard for the quality of sugar products. The results showed that the laboratory-produced GM sugar was pure enough and representative for this study.

Molecular analysis results differed from those obtained by Joyce in 2013 (Joyce et al., 2013), who stated that only the leaves, stems, fibers, and juices from transgenic sugarcane can be characterized using traditional PCR. The present work revealed that in addition to these products, other materials, namely, filter mud, syrup, molasses, and white granulated sugar derived from GM sugarcane, can be distinguished from those derived from non-GM sugarcane through RT-qPCR. DNA from leaves, stems, fibers, and juices can be extracted easily with the CTAB method. Clarified juice retained few residual plant cells, and normally the remained DNA in it was complete without degraded. Thus, DNA from juices can be extracted easily and sampled for molecular analysis. Filter mud is an impurity generated by the heating and concentration of clarified juice. Most residual plant cells remaining in juices were centralized, filtered out, and converted to filter mud, which contains substantial residual plant cells. Although some of the cells may have been destroyed and DNA may have been degraded to a certain degree because of heating treatment, the filter mud still contained sufficient DNA for extraction and molecular analysis. After the filtration treatment, the plant cells remaining in the syrups were considerably reduced but not completely eliminated. However, consistent heating destroyed most of the cells, and DNA was degraded. Thus, a large amount of syrup should be sampled for the enrichment of residual DNA fragments for molecular analysis. Molasses is segregated from the crystallization of white granulated sugar and accumulate the residual cells in the syrup. Thus, molasses contained more plant cells and DNA than the syrup in each sample. However, consistent heating treatment destroyed most of the cells and degraded the DNA. Thus, many molasses samples are needed in DNA enrichment for molecular analysis. White granulated sugar is crystallized during the heating and concentration of syrup and becomes a purified product with few residual DNA fragments. DNA enrichment for molecular analysis requires a large amount of white granulated sugar. In the present study, we sampled 5 ml of syrup, 5 ml of molasses, and 5 g of white granulated sugar (GM and non-GM sugar) for DNA extraction. A small amount of DNA model was obtained for molecular analysis.

In molecular analysis, RT-qPCR was more reliable than traditional PCR. Most DNA fragments extracted from the syrups, molasses, and white granulated sugar were poorly degraded and extremely short. Primers that amplify short products should be designed for amplification. RT-qPCR is preferred in short-fragment amplification, because its result is evaluated based on the CT value and dissociation curve, rather than on the agarose gel. Short product fragments, especially those inefficient for amplification and produce only a few products, cannot be easily observed on agarose gel.

ELISA results indicated that Cry1Ab and EPSPS proteins were present in the leaves, stems, fibers, and juices but not in the filter mud, syrups, molasses, and white granulate sugar derived from the GM sugarcane because the latter group consisted of heat-treated materials. This result is in accordance with the study of Joyce (Joyce et al., 2013).

The results of toxicity assay on GM and non-GM sugar showed that the GM sugar was nontoxic to the larvae primarily because of its lack of active Cry1Ab protein. This result is also in accordance with the ELISA results.

Traditional PCR or RT-qPCR results showed that the leaves, stems, fibers, and juices derived from transgenic sugarcane can be distinguished from those derived from nontransgenic sugarcane. In addition, filter mud, syrups, molasses, and white granulated sugar from transgenic sugarcane can be distinguished from those derived from nontransgenic sugarcane. Highly efficient DNA extraction methods should be used in enriching DNA models for PCR amplification and obtaining accurate results from molecular analysis. The findings indicated that although residual DNA was found in all derived materials, active proteins were absent in the filter mud, syrups, molasses, and purified white granulated sugar. Thus, transgenic sugarcane can be commercialized and used in producing GM white granulated sugar.

## Data Availability Statement

The original contributions presented in the study are included in the article/supplementary material, further inquiries can be directed to the corresponding authors.

## Author Contributions

WZW take charge of the operation of the entire research and paper writing. BPY and ZDW take charge of the producing of GM sugar. XYF and LBS helped to finish the ELISA experiment. JGW and TTZ helped to finish the toxicity feeding bioassay. QNW helped to finish the Quality analysis of GM sugar. SZZ and ZQM are the directors of the original research and paper writing. All authors contributed to the article and approved the submitted version.

### Conflict of Interest

The authors declare that the research was conducted in the absence of any commercial or financial relationships that could be construed as a potential conflict of interest.
